# Experiencing your brain: neurofeedback as a new bridge between neuroscience and phenomenology

**DOI:** 10.3389/fnhum.2013.00680

**Published:** 2013-10-24

**Authors:** Juliana Bagdasaryan, Michel Le Van Quyen

**Affiliations:** ^1^Centre de Recherche de l’Institut du Cerveau et de la Moelle Epinière, INSERM UMRS 975 - CNRS UMR 7225, Hôpital de la Pitié-SalpêtrièreParis, France; ^2^Université Pierre et Marie CurieParis, France

**Keywords:** neurophenomenology, neurofeedback, multiscale neural dynamics, downward causation, voluntary action

## Abstract

Neurophenomenology is a scientific research program aimed to combine neuroscience with phenomenology in order to study human experience. Nevertheless, despite several explicit implementations, the integration of first-person data into the experimental protocols of cognitive neuroscience still faces a number of epistemological and methodological challenges. Notably, the difficulties to simultaneously acquire phenomenological and neuroscientific data have limited its implementation into research projects. In our paper, we propose that neurofeedback paradigms, in which subjects learn to self-regulate their own neural activity, may offer a pragmatic way to integrate first-person and third-person descriptions. Here, information from first- and third-person perspectives is braided together in the iterative causal closed loop, creating experimental situations in which they reciprocally constrain each other. In real-time, the subject is not only actively involved in the process of data acquisition, but also assisted to directly influence the neural data through conscious experience. Thus, neurofeedback may help to gain a deeper phenomenological-physiological understanding of downward causations whereby conscious activities have direct causal effects on neuronal patterns. We discuss possible mechanisms that could mediate such effects and indicate a number of directions for future research.

## FIRST AND THIRD: THE NECESSARY CIRCULATION

The major research domains in cognitive neuroscience aim to characterize human experience, mind, and consciousness. By randomization, standardization procedures and statistical analysis, this approach seeks to extract the most essential invariant mechanisms, generalizable to the entire population. However, it is curious that in the study of necessarily subjective phenomena of mental processes, we refuse to consider them as such. Instead of elaborating on the subjectivity, we are paradoxically disregarding the most characteristic feature of our mind. In the mid-1990s, Varela (1996) proposed a scientific program termed “Neurophenomenology,” conceptualized as a remedy for the hard problem of consciousness (Chalmers, 1995). Rather than studying the hard problem *per se*, this proposal was of pragmatic nature, oriented toward the explanatory gap of how to relate neurobiological and phenomenological features of consciousness. Neurophenomenology encourages a combined investigation of scientific observation and subjective experience in scientific research, without denying the necessity of a rigorous methodological approach in the acquisition of first-person data. The dialog between the two different types of data generation is considered to result in a twofold profit:

(1) Phenomenologically enriched neural data make ongoing mental or physical processes accessible to the subject that would otherwise remain unconscious. New variables might be opened up for personal observation and introspection.
(2) The neuroscientist is guided by the subjective report, which provides a strong constraint on the analysis and interpretation of physiological data relevant to conscious experience. Relating physiology to phenomenology is expected to uncover subtle details in neural data by means of the phenomenological perspective.

In that way, mutual constraints given by the complementary perspectives enable the specification of our models of phenomenology, and the associated neural activity.

As evidenced by this special issue, Varela’s call has not gone unanswered, and recent years have seen the development of a small but growing literature exploring the interface between phenomenology and neuroscience. The emergence of the field of neuropsychoanalysis (Panksepp and Solms, 2012) attests to this trend, in addition to the increasing number of studies including both qualitative and quantitative data as on visual perception (Lutz et al., 2002), lucid dreaming (Hobson, 2009), the initiation of epileptic seizures (Le Van Quyen and Petitmengin, 2002) or the recent study elucidating cognitive processes that correspond to the default mode network activation (Garrison et al., 2013).

However, the integration of first-person data into the experimental protocols of cognitive neuroscience still faces a number of challenges. Two major methodological concerns regarding the quality of the first-person data are that (1) subjective reports can be untruthful or lacking precision, and (2) experience might be changed by the very fact of reporting. From the epistemological perspective it is not evident how to relate the qualitative and quantitative data in methodologically valid and meaningful ways (Lutz and Thompson, 2003).

Although valuable work has sharpened the acquisition methods of qualitative data (Lutz et al., 2002; Depraz et al., 2003; Petitmengin et al., 2007), a meaningful link between these and the neural data remains challenging. The central difficulty is the temporal scale of neural and subjective events. While many neural events can last only a few hundreds of milliseconds, the temporal resolution of thought and memory processes are at a coarser scale of seconds. The approximate sense of personal timing will thus limit the precision of an oral report. Moreover, subjective reports are usually obtained either in intermittent periods or at the end of the experiment, but never in a concurrent manner with neural data. Because the acquisition of data occurs independently for each, the reports and the recordings can merely be compared or correlated *a posteriori*. Since the precision in the temporal dimension is a crucial variable for neural processes, the long delay introduced between the experience and the corresponding neural activity will significantly reduce the amount of information that can be extracted from such comparisons. When the personal account is supposed to guide analysis and interpretation of neural data, a causal link between the perspectives seems necessary. Similarly, in order to benefit from neural data for deeper introspection, temporal contingency between personal perception and neural events is essential, as was shown in associative learning (Sulzer et al., 2013a).

Given these limitations of the neurophenomenological approach, an experimental procedure that would facilitate a more direct mapping of neural and personal data is desirable. We propose that the paradigm of neurofeedback is a good candidate to yield further progress in the field. The idea to unify first-person and third-person data is at the very core of neurofeedback, making it appropriate for studies within the research program of neurophenomenology.

## NEUROFEEDBACK – THE PAST AND THE PRESENT

**FIGURE 1:**
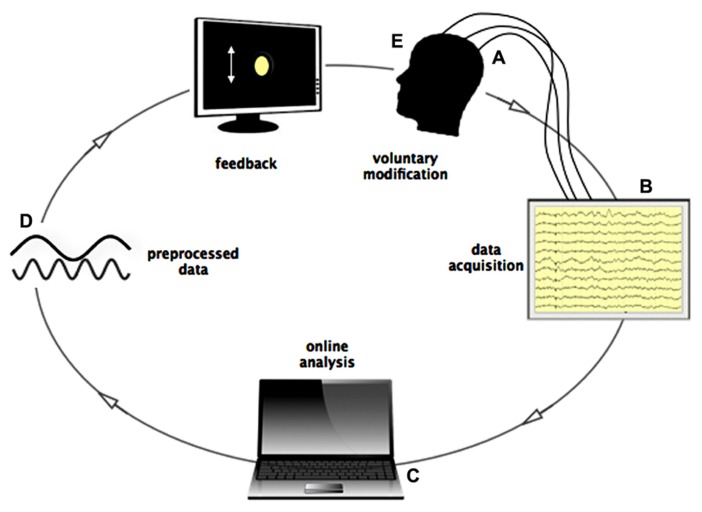
**Loop of online data streaming during Neurofeedback.** **(A)** Signals from scalp-, macro-, and/or microelectrodes are pre-amplified locally and sent to the acquisition system. **(B)** All electrodes are recorded and stored on the local computer. **(C)** Data is read by another device, where online analysis is performed (frequency filtering, spike detection, spike sorting) in time bins of 0.5 s. **(D)** Processed data is presented to the subject in form of a graphical, moving object, or sound changing in frequency according to the recorded activity. **(E)** Subject controls the graphical object by influencing his brain activity through subjective experience.

If provided with real-time feedback, human, and animal subjects can learn to control various measures of their own bodily and neural activity such as heart rate, skin conductance, the Blood-Oxygen-Level-Dependent-(BOLD) response, the oscillatory activity, and even the spiking of single cells (Fetz, 1969, 2007; Evans, 2007; Cerf et al., 2010; Roelfsema, 2011). Based on brain electrical signals transmitted in real time, inner control of one’s own neuronal activity may be learned with the aid of a brain-computer interface, which serves to preprocess and display a person’s instantaneous brain activation on a computer screen through what is known as a “neurofeedback” loop. This visual display behaves like a virtual “mirror” to real electrical activities produced by the cerebral cortex. For example, using neurofeedback of electroencephalographic (EEG) signals, the power of participants’ neuronal oscillations in a given frequency (e.g., the alpha band from 8 to 12 Hz) are visually displayed to them, typically in the form of a bar graph whose height is proportional to the real-time EEG amplitude and which fluctuates accordingly (**Figure 1**). Participants try to learn to manipulate this visual feedback, increasing/decreasing it to a predefined threshold level, with a reward when amplification/suppression to this threshold is achieved. Guided by the visual feedback process, the participant can search for a relationship between the conscious experience and the changes in neural data in ongoing data streaming.

The pioneering studies in the field of neurofeedback were conducted as early as the 1960s starting with the important work by Fetz (1969) on primates, showing the operant conditioning of single cell spike trains in the motor cortex. The motor cortex is probably the most obvious place to search for a cortical signal directly associated with volitional movement (Libet et al., 1983; Haggard et al., 2002; Fetz, 2007). This may be one of the reasons why a substantial part of neurofeedback research was conducted on paralyzed or locked-in patients recognizing the need of people with disabilities, aiming to restore their communicative or motor functions. Brain-computer interfaces were tested in amyotrophic lateral sclerosis, brain stem stroke, or spinal cord lesions using signals including slow cortical potentials, P300 potentials, and alpha or beta rhythms recorded from the scalp, and cortical neuronal activity recorded by implanted electrodes (Wolpaw et al., 2002; Birbaumer and Cohen, 2007; Jackson and Fetz, 2011). The successful cases in these applications encouraged the usage of neurofeedback for other neurological and neuropsychiatric conditions. Subsequently, positive neurofeedback effects were achieved in substance addiction (Sulzer et al., 2013b), Attention-Deficit-Hyperactivity-Disorder (ADHD; Gevensleben et al., 2009), autism spectrum disorder (Kouijzer et al., 2010), emotional regulation (Johnston et al., 2010), Parkinson’s disease (Subramanian et al., 2011), and epilepsy (Kotchoubey et al., 2001; Nagai, 2011).

The starting point in most of these studies was a predefined physiological profile of a certain function or pathology to be enhanced or counterbalanced through neurofeedback. As for example in a study on autism, the success of the neurofeedback training was due to the decrease of the excessive theta power (4–8 Hz) in the anterior cingulate cortex, known to be involved in social and executive dysfunctions in autism (Kouijzer et al., 2010). Beside clinical application, the effects of neurofeedback training were also explored in general cognitive functions. Improved mental rotation, perceptual learning, episodic memory, and higher intelligence scores were reported after training (Hanslmayr et al., 2005; Keizer et al., 2010a,b; Shibata et al., 2011; Zoefel et al., 2011).

A particularly interesting approach consisted of using intracranial EEG recorded in epileptic patients to design a simple computer interface (also called “Brain TV,” ; see Petitmengin and Lachaux, 2013) and to display to patients in real-time their activity recorded at particular cortical locations in several frequency bands, including alpha (8–12 Hz), beta (12–30 Hz), and gamma bands (>40 Hz; Lachaux et al., 2007). During such neurofeedback sessions, the patients were able to observe their own neural data. Once they have identified a possible link between their acts and the signal response (e.g., by solving arithmetic exercises or relaxation) subjects were able to deliberately control the brain activity (Lachaux et al., 2007). In most of the discussed studies a conscious, cognitive strategy was adopted to find a link between inner events and the corresponding neural signal (e.g., expressing an emotion, performing mental imagery, building up an intention, remembering an event, or other cognitive acts; deCharms, 2008). However, an implicit type of successful learning akin to skill learning has also been discussed, emphasizing the role of the subcortical motor system (Birbaumer et al., 2013). The hypothesis that brain-self-regulation can be achieved without a high cognitive, explicit, and conscious strategy is supported by animal studies on primates and rodents making use of associative learning or operant conditioning (Fetz, 1969; Koralek et al.,2012).

The modulation of a specific physiological substrate appears to be dependent on the sensory feedback provided to the subject. As several studies have demonstrated, the control over rt-fMRI brain activation was trainable with proper and not sham feedback (Sulzer et al., 2013b). One study that confirms that feedback is necessary information for self-regulation comes from a study on chronic pain patients showing that the feedback of neural activity was necessary for them to succeed in controlling the neural processing behind pain perception reducing perceived pain. One would assume that pain patients already have continuously available sensory feedback of their personal pain level, as well as a strong motivation to restrain the pain intensity (deCharms et al., 2005). Nevertheless, the personal pain perception alone was not sufficient for the control of pain, whereas the feedback on neural activity seemed to provide additional information that played a crucial role in the ability to control physiological processes.

Overall, these studies indicate that control over neural activity is not confined to a particular neurophysiological function or a specific anatomical location. Rather, it seems to be a more general property of the brain that can be learned for different neural profiles and various clinical or cognitive conditions given appropriate feedback.

## NEUROPHENOMENOLOGY MEETS NEUROFEEDBACK

### REAL-TIME LOOP BETWEEN FIRST-PERSON AND THIRD-PERSON DATA

Neurofeedback offers a way to relate the phenomenological structure of subjective experience with a real-time characterization of large-scale neural operations in a continuous manner over the course of the experiment. In the setup, the current state of neural activity, reflecting moment-to-moment changes in perception and cognition of the subject, is recorded at multiple cortical sites. After processing, the neural variable is presented to the subject with a delay of no more than 0.5 s. The subject is asked to monitor all mental acts or changes in personal experience that could correspond to the fluctuation of the signal. While trying to detect the link between the two, the subject’s principal task is to guide mental activity such that the neural signal reaches an upper or lower threshold. With this task in mind, the subject is continuously monitoring whether a change in the mental process is associated with a change in the recorded signal in the desired direction. By such deliberate manipulation of the signal, the subject enriches the neural data with ongoing personal experience, shaping his or her own brain activity. In the same way, the scientifically presented data can influence the subject, when upon the subsequent iteration of data streaming (next 0.5 s), the outcome of the scientific analysis might make the subject change his or her approach. The loop between the subject and the data becomes causally bidirectional.

In this way, online information of physiological variables allows the subject to gain access to a neural process that is related to the mental activity, which is usually hidden from awareness. The constant feedback facilitates monitoring of neural control and allows the subject to evaluate the efficacy of the chosen strategy (e.g., remembering moments from childhood) regarding the overall task. Through practice across the sessions of a training period, continuous introspective effort promotes insights on arousal, concentration, distraction, self-awareness, and self-regulation. Gradually, an understanding of the link between the change in cognition and its neural correlate emerges, which is refined on a trial-and-error basis, until it can be systematically exploited in a reliable way. The subject learns to control several electrodes at various cortical sites, tries to modulate different oscillatory frequency ranges, spiking activity, or synchronization degrees. Ultimately, the subject is capable of selecting which electrode responds best to the voluntarily induced mental events and which frequency range or other parameter is the easiest to modify.

### CO-DETERMINATION BETWEEN FIRST-PERSON AND THIRD-PERSON DATA

The inherent feature of this setting is the mutual constrain between phenomenology and neuroscience. Because information from first- and third-person perspectives are united and co-determine each other in the iterative loop of real-time neurofeedback, the epistemological concern of how to relate neural and personal data is resolved. A meaningful link between subjective and neuroscientific data is created through this causal relationship, which offers a guideline for data analysis and interpretation. Moreover, as discussed in Section “Neurofeedback – The Past and the Present,” it is difficult to achieve a simultaneous sampling of subjective experience in parallel to the acquisition of neural data without a significant delay. Neurofeedback is advantageous in this respect because subject is embedded in the experimental setting, allowing a new real-time dimension for data correspondence. Because the first-person data is included in the overall data stream, no back-and-forth switch is required between objective and personal data. An additional strength is that the methodological problem of an untruthful, imprecise or biased report can be circumvented. Although oral or written subjective descriptions may still be useful to elucidate the best cognitive strategy, they are not strictly necessary for the realization of the neurofeedback paradigm.

## A PHYSIOLOGICAL DESCRIPTION OF NEUROFEEDBACK

An understanding of physiological factors underlying neurofeedback would not only uncover the mechanisms relevant for volitional modulation of neural processes but also advance our possibilities to therapeutically adapt neurofeedback training to different clinical conditions. Our knowledge of the neural substrates underlying neurofeedback is limited. However, an important indication comes from above mentioned studies revealing the fact that neural control is most efficiently initiated by a cognitive strategy demanding attentional processes (although see Birbaumer et al., 2013 for a different perspective). This observation exposes the link between high-level cognitive activity and the changes in dynamics of brain activity implying that top-down effects on conscious mental events play an important role during neurofeedback. In the following, we aim to characterize a general relationship and codetermination between neural and mental events, which would allow us to formulate a potential mechanism of neurofeedback.

### TOP-DOWN PROCESSING AND DOWNWARD CAUSATION

**FIGURE 2:**
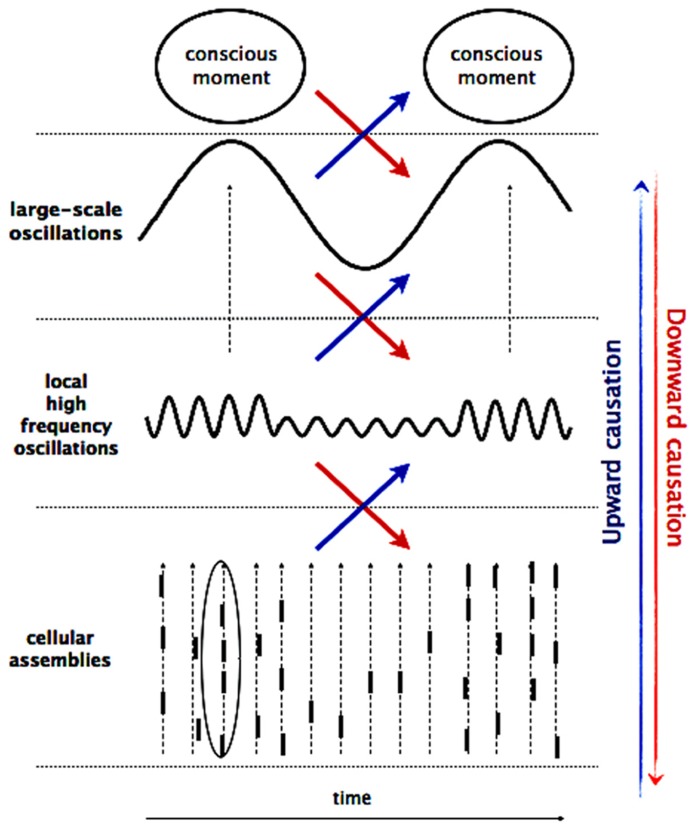
**Multiscale interaction.** The macro-, meso-, and microscopic processes are braided together by co-occurring multifrequency oscillations, giving rise to upward and downward causation. Activity at micro-scale (cellular assemblies) sums up to local activities at meso-scale, which in turn gives rise to large-scale dynamics and result in a conscious event. In opposite way, cognitive effort influences global brain oscillations in the low- frequency range, which constrain local oscillations in the high-frequency range by variations of the underlying neuronal excitability. These high-frequency oscillations determine the probability of occurrence of spikes and their temporal coincidences on the millisecond scale.

It is widely accepted that neural processes crucial for consciousness (i.e., perception and cognition) rely on the transient and ongoing orchestration of large-scale assemblies that comprise neuronal populations in widespread networks of frontal, parietal, and limbic areas. As proposed previously (Varela, 1995; Varela et al., 2001; Le Van Quyen, 2003), such large-scale assemblies constitute a fundamental self-organizing pole, exerting a “driving” effect on multiple neuronal activation levels at macro-, meso-, and microscopic scales and providing a valuable physiological candidate for the emergence and the flow of cognitive-phenomenal states (**Figure 2**). Numerous studies, using unit recordings or functional imaging, have established that there are bi-directional causal relationships between multiple spatial and temporal scales where on one hand, activity on a lower scale gives rise to an emergent phenomenon and on the other hand, the large-scale patterns have the potential to re-influence the small-scale interactions that generated them (Fröhlich and McCormick, 2010; Anastassiou et al., 2011; Buzsáki et al., 2012). In order to stress their active efficacies, these bottom-up and top-down interactions are often referred to as upward and downward causation (Campbell, 1974; Thompson and Varela, 2001).

In this context, there is increasing evidence that brain oscillations play a key role in mediating these multi-scale communications (Fries, 2005; Le Van Quyen, 2011). As a general rule, lower frequency oscillations allows for an integration of neuronal effects of longer duration and larger areas of involvement (Penttonen and Buzsaki, 2003). In contrast, high-frequency oscillations tend to be confined to small ensembles of neurons and facilitate a temporally more precise and spatially limited representation of information. Consequently, slow cortical oscillations lead to cyclical modulations in neuronal excitability that determines whether faster local oscillations or neuronal discharges are attenuated or amplified (so called cross-frequency coupling). Consistent with this idea, recent data confirmed that attention modulates the phase of delta activity (1–4 Hz) in the visual cortex, which in turn modulates the power of higher frequencies and the firing of neurons (Lakatos et al., 2008). It was also shown that slow frequency activity in 4–7 Hz range recorded in the local field potential can predict the higher frequency (30–200 Hz), as well as single unit activity (Buzsaki and Draguhn, 2004; Canolty et al., 2006; Jensen and Colgin, 2007). At a lower spatial scale, top-down effects can influence spike-field locking, promoting spikes synchronization to preferred oscillatory phases (Womelsdorf et al., 2007; Rutishauser et al., 2010; Engelhard et al., 2013). Furthermore, hierarchical interactions between areas appear to be specific to the direction of information processing. For example, it was shown that top-down and bottom-up effects between frontal and parietal cortices take effect through synchronization on different oscillatory frequency ranges (Buschman and Miller, 2007; Knight, 2007).

Given the relationship between the multiple scales as manifested in different oscillatory rhythms, a potential neurophysiological mechanism underlying neurofeedback function can be hypothesized from these considerations on downward causation: during neurofeedback, higher cognitive functions such as monitoring or introspection are required, which involve a large number of subprocesses and thus, they recruit neural assemblies over extended regions. Changes in large-scale neural activity are therefore expected and should be detectable in low frequent oscillatory activity. In turn, following the rule of cross-frequency coupling, these changes are mediating downward influences via the precise temporal windows of integration imposed by oscillatory activity, giving rise to effective communication between distributed networks and regulating the flow of information processing.

Thus, in this scenario an initial large-scale activity triggered by cognitive effort can percolate down to the small scale of single neurons, where overall dynamics are tied together by co-occurring oscillations in different frequency ranges inducing changes in neuronal excitability. Importantly, although the conceptualization of neural control is based upon downward causation, physiologically, top-down, and bottom-up effects are reciprocally defined and contingent on each other. These effects are distinguished conceptually and can be empirically quantified separately. However, the physiological existence of these two types of causalities between neural and mental events cannot be dissociated.

### TESTABLE HYPOTHESES

**FIGURE 3:**
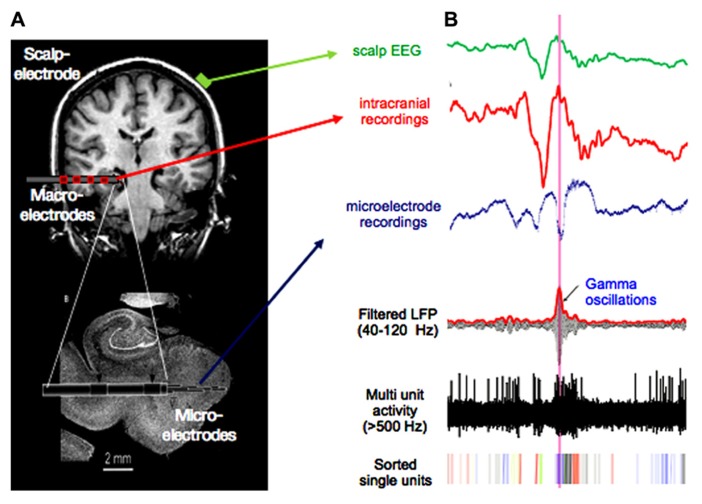
**Multiscale recordings.** **(A)** Scalp-electrode (green), clinical multi-contact macro-electrode (red), and micro-electrode emerging from the tip of the macro-electrode (resolution: volume <1 mm^3^ on a millisecond scale). Such recording setups are used for presurgical evaluation in epilepsy. **(B)** Signal from scalp-, macro-, and micro electrode in green, red, and blue, respectively. Lower three traces show micro-electrode recordings filtered in the gamma band, with applied high-pass filter above 500 Hz and sorted spikes for different neurons. Note the high-frequent activity present in the micro-electrode recording, which is not visible in the signal from macro- or scalp-electrodes.

The model proposed here attempts to integrate the evidence for neurofeedback control with the view of multi-scale coordination in neuronal dynamics that has emerged during recent years. The advantage of this model is to derive concrete testable hypotheses. Notably, we expect that a multiscale approach with data recorded on multiple spatial scales leads to greatest insight because investigation of the coupling between the multiple spatiotemporal scales is possible. Such data can be, for example, obtained from patients with drug resistant epilepsy undergoing long-term monitoring, where scalp, depth, and micro electrodes (Fried et al., 1997; Le Van Quyen et al., 2010) are used for simultaneous data recording (**Figure 3**). This approach combining single cell recordings with a global monitoring of large-scale brain activities has the potential to reveal regional diversity in the properties of local brain activities such as their spatial topography, spectral characteristics, propagation, and phase coherence (Lachaux et al., 2003). It allows us to distinguish the global, local, and high-frequency processes, and their interactions, that constitute elementary information processes. Local field potential measurements combined with recording of neuronal discharges will provide us with information about the cooperating inputs onto the recorded cell population.

Using such data, the aim is to find the physiological markers of neural control. In the search for characteristics of successful neurofeedback, the examination of successful trials, preceding intervals, and the contrast to failed trials is the thread of the analysis. A systematic record of key parameters such as power, amplitude, synchronization, or phase locking reveals changes across sessions and facilitates tracing the evolution of important factors over the course of the training period. Thereby, a shift in parameters between the first and the last sessions may not necessarily be progressive or linear.

One important question is to determine at what spatial and temporal scale the neural dynamics can be influenced in most efficient manner. In **Figure 3**, scalp-, intracranial EEG, and micro-electrode recordings display components in different frequencies that are characteristic for each data type. In the scalp-EEG slow rhythms are predominant, whereas the micro-recordings contain much faster spiking activity. These data, simultaneously recorded and filtered in corresponding bands, can be successively used as feedback within the brain-computer-interface for a comparison of success rates as well as required training time. According to the presented model and the evidence reviewed earlier, we suggest that effective neurofeedback can best be achieved at the macroscopic level, by the voluntary control of cortical slow oscillations. In particular, we propose that these large-scale waves mediate downward influences via a precise temporal patterning of local processing and provide a vehicle for top-down control of local high-frequency oscillatory activity and on firing rate at the single cell level.

Due to anatomical and organizational differences of the brain, it is likely that these modulations will not be homogeneously efficient across cortical regions. Some dynamical features may be more beneficial for top-down effects than others. Will the control be best achieved on areas that are known to be hierarchically structured including recurrent and feedback pathways and thus appropriately wired for top-down control, such as the motor, visual, or other primary sensory cortices? Is neural control in temporal areas, hippocampus, amygdale, and prefrontal cortex also possible and if so, does it take longer to acquire sufficient regulation?

Scharnowski et al. (2012) have shown generalization effects of improved perceptual sensitivity through neurofeedback training across stimuli and tasks. Can trained effects be potentially generalized across electrode locations or frequency bands? Spatial generalization may be possible when structural and dynamical organization of cortical sites is sufficiently similar, so that the same cognitive strategy can become operative. To some extent this can be anticipated by examining dynamical features of the signal such as predominant frequency ranges or firing rate baselines and patterns. In contrast, generalization across frequency bands might be predictable with measures of cross-frequency coupling. When high degrees of amplitude-phase coupling (nested oscillations) are present, a frequency range that has not been the direct object of neurofeedback training is likely to be influenced by the same strategy when tested directly. One other type of generalization might occur in conditions without neural feedback. It is plausible that once neural control is reliably trained, it can be retrieved implicitly by exploiting the proven strategy even without sensory feedback. This can be for example tested with a transfer session at the end of neurofeedback training.

Another crucial question is whether cellular plasticity is taking place during neurofeedback, which may serve to regenerate motor functions or boost memory processes (Seitz, 2013). When during successful neurofeedback the signal at the conditioned electrode spreads along existing network connections and propagates from the electrode position to more distant sites, over time, synaptic connections between simultaneously recruited neurons are strengthened. This can be tested by exploiting micro-electrodes for training and analysis. Correlated spiking behavior as well as the convergence toward the same preferred spiking phase between adjacent micro-electrodes may be indicative of this process. If plasticity is occurring, these variables should shift and remain different from baseline even during spontaneous intervals as compared to values prior to training.

Finally, an unresolved issue to be addressed with future studies is the difference between responders and non-responders to neurofeedback. Is the full variance explained by varying skills of introspection or are there detectable dynamical differences in neural data? For example, the investigation of individual predominant frequency ranges in the spectrum could be indicative of necessary dynamic components.

## GENERAL COMMENTS AND FUTURE PERSPECTIVES

The prime concern of future neurofeedback studies is, with the subjects’ help, to identify the principles and mechanisms behind neural control. Pursuing a neurodynamical approach, we believe that electrophysiological data sampled at several spatial scales is appropriate to reveal the mechanisms behind willful modulation of neural activity during neurofeedback training, as discussed in Section “A Physiological Description of Neurofeedback.” The distinct strength of a multiscale approach is that it allows us to test hypotheses derived from consideration of top-down effects and downward causation.

At the second stage, the obtained generic description of physiological factors that mediate willful regulation would be the vehicle for all further application of the neurofeedback technique, specifically designed to best affect the desired structures or processes (as in depression, ADHD, or other conditions). When a certain function needs to be regulated, firstly, it is essential to know how it is neurally encoded. Therefore, at this point our knowledge about the substrate of neurofeedback as well as the cognitive profile in question needs to be combined to design an optimal experimental protocol in order to maximize the efficiency of the training. Although neurofeedback can be applied to a condition on which we have only limited insight, in general, knowing the target mechanisms will increase the efficiency of the neurofeedback training.

Beside the relevance of studies using invasive intra-cortical recordings, scalp-EEG and fMRI studies are also indispensable for promoting non-invasive use of neurofeedback in the general population. A particularly promising approach is the combination of rt-fMRI recordings with decoding techniques (Scharnowski et al., 2012), that could be of great use for clinical applications in locked-in and paralyzed patients. Depending on the chosen methodology, initial assumptions of the technique need to be considered. The working hypothesis when using rt-fMRI is based on the metabolism of neuronal activity and the derived BOLD response in precise brain areas related to a given cognitive function (deCharms, 2008; Scharnowski et al., 2012). In contrast, the work done with EEG derives from the assumption of a temporal coding through oscillatory activity (Engel et al., 2001). Rt-MRI focuses on the change in specific brain structures, whereas the precise temporal character of the EEG signal promotes control of diffuse, global oscillatory processes in various frequency bands. Both methods can be desirable in a given context, but their features have to be carefully considered when designing the experimental setup.

As seen from previous studies of neurofeedback, application of neurofeedback can be wide-ranging. In the case of epileptic patients, neurofeedback training can consist of dampening epileptic activity in pathological regions (e.g., by perturbing local dynamics with a dominant theta rhythm) as to reduce seizure frequency or intensity. Another intriguing application of neurofeedback is in schizophrenia, where impaired neural synchronization in gamma and beta ranges, but not in lower frequencies, was shown (Uhlhaas and Singer, 2006, 2010; Uhlhaas et al., 2008). For this profile, the neurofeedback could target the synchronization in these frequency bands directly or indirectly through the theta band via cross-frequency coupling. One area of agreement in depression research is the hyperactive stress-response of the hypothalamic-pituitary-adrenal (HPA) axis, whose activity is controlled by functional axes including the hippocampus and the amygdala. The activity in these two structures is reduced and enhanced in depression, respectively (Nestler et al., 2002) and could be potential training parameters in neurofeedback. Finally, enhancing attentional processes might be worthwhile considering not only in ADHD-children, but in a healthy population in general. Cholinergic inputs originating in basal forebrain were discussed as crucial components of the network mediating sustained attention (Sarter et al., 2001; Deco and Thiele, 2011). Through neurofeedback induced plasticity in localized cortical sites (Koralek et al., 2012; Scharnowski et al., 2012) long-term changes can strengthen the projections of cholinergic neurons to boost reading skills or the ability to stay in focus.

From the phenomenological perspective, further improvements can be made to integrate personal accounts within neural data. The major task is to support the subject in the process of introspection and self-discovery to achieve control over neural activity. Despite the fact that ongoing subjective information is accessible only to the subject, it is possible to assist the subject by asking for an ordinary report offline, between training sessions. For instance, the interviewing techniques used to anticipate the seizures (Petitmengin et al., 2007) represent a valuable tool, when guiding subjects to a more refined perception. Meditation techniques can also be used to instruct patients to refine skills of self-observation and self-perception (Garrison et al., 2013). Alternatively, depending on the spontaneous success, a more standardized approach to assist the subject can consist of proposing to engage in cognitive tasks that are known to activate a specific cortical site or neural processes. Another possible strategy is to instantaneously reward the subject for successful control. This can help to set a temporal marker, creating a clear contingency between the experience and the changes in the display. Eventually, this can result in the ability to implicitly distinguish between noise and indicative signals (Sulzer et al., 2013a). Such markers can be, for example, represented in the form of graphical tokens on the screen.

## CONCLUSION

In the present article, we have proposed that neurofeedback is an appropriate experimental paradigm to bridge the gap between neuroscience and personal experience. Unlike other bodily organs that allow us to process sensory information of a certain modality, humans lack a faculty to experience their ongoing brain activity. The technical and experimental setups of neurofeedback create an interface between scientific and personal data types such that both are embedded in one information stream. This provides the subject a window to experience his or her own neural activity, which has proven to carry useful information in the context of self-regulation. Such a setting combines seamlessly with the dynamical systems idea proposed by Varela in the “enactive” approach (Thompson and Varela, 2001), where the organism both initiates and is shaped by the environment (Varela et al., 1991). Thus, neurofeedback experimentally implements the notion of an autonomous organism that is literally “self-governing” its neural dynamics and cognition by means of interaction between the environment (sensory feedback of brain activity) and the organism (personal experience). A concrete application of the enactive theory involving the subject’s contribution allows neuroscience to study how the process of mutual specification and selection between brain and mind is taking place. The real-time dimension provided by neurofeedback facilitates the on-line comparison of data sources without a significant delay, which methodologically reconciles personal and neural data. In that, we emphasized the relevance of understanding neural signatures of successful voluntary self-control that are probably mediated by hierarchically organized neural processing. Identifying electrophysiological markers of neurofeedback and its evolution is therefore a major objective for future studies.

The benefit for phenomenology and science is mutual. Psychologically, the ability to self-regulate processes correlated to mental experience cannot be underestimated (Christoff et al., 2011). The subject’s introspection is trained over time, giving him or her a better sense for self-awareness and self-control. This can change the self-image, empowering the subject to a greater self-determination, especially valuable in developing personalities and certain clinical conditions.

Altogether, the global perspective of neurofeedback has far-reaching implications: the capacity to voluntarily modulate physiological functions can yield control over various neural mechanisms of cognition and behavior. Such a tool for self-regulation can assist us to achieve a better self-awareness, self-knowledge, and enhanced cognitive skills. In addition, neurofeedback has proven clinical benefits. If one can learn to regulate particular brain regions, or induce specific neural patterns, in the long term we may obtain an alternative method to treat diseases in a non-invasive, introspective way.

## Conflict of Interest Statement

The authors declare that the research was conducted in the absence of any commercial or financial relationships that could be construed as a potential conflict of interest.
